# MIR205HG acts as a ceRNA to expedite cell proliferation and progression in lung squamous cell carcinoma via targeting miR-299-3p/MAP3K2 axis

**DOI:** 10.1186/s12890-020-1174-2

**Published:** 2020-06-08

**Authors:** Limin Liu, Yulei Li, Ruifang Zhang, Chun Li, Jing Xiong, Yuan Wei

**Affiliations:** 1 Respiration Department, Tianyou Hospital Affiliated to Wuhan University of Science and Technology, No.9, Tujialing, Wuchang District, Wuhan, 430064 Hubei China; 2 Three Wards of Outpatient Service, Wuhan Jin Yin Tan Hospital, No.1 Yintan Road, Dongxihu District, Wuhan, 433013 Hubei China

**Keywords:** Lung squamous cell carcinoma, MIR205HG, miR-299-3p, Competing endogenous RNA (ceRNA), MAP 3 K2

## Abstract

**Introduction:**

Long noncoding RNAs (lncRNAs) have been associated with many types of cancers, but their molecular mechanisms in lung squamous cell carcinoma (LUSC) have not been fully studied. Therefore, the current study investigated the regulation role of microRNA-205 host gene (MIR205HG) in LUSC and recognized the target genes managed by this lncRNA.

**Methods:**

MIR205HG expression was assessed by the quantitative real-time polymerase chain reaction (qRT-PCR) analysis. The effects of silenced MIR205HG on cell biological behaviors were detected by colony formation assay, transwell assay, flow cytometry analysis and western blot analysis. Luciferase reporter assay and RNA immunoprecipitation (RIP) assay were utilized to proof the binding relationship between miR-299-3p and MIR205HG/mitogen-activated protein kinase kinase kinase 2 (MAP 3 K2).

**Results:**

The expression levels of MIR205HG in LUSC tissues and cell lines were obviously up-regulated. Down-regulation of MIR205HG expression remarkably reduced cell proliferation, migration and epithelial-to-mesenchymal transition (EMT) progression, whereas promoted cell apoptosis. MIR205HG could bind with miR-299-3p and down-regulation of MIR205HG elevated miR-299-3p expression. MAP 3 K2 acted as the target gene of miR-299-3p and was up-regulated by MIR205HG overexpression. Overexpressing MAP 3 K2 could counteract the effects of down-regulating MIR205HG on LUSC progression to some degree.

**Conclusion:**

MIR205HG acts as a competing endogenous RNA (ceRNA) to expedite cell proliferation and progression via targeting miR-299-3p in LUSC.

## Introduction

Lung cancer maintains a serious common reason of tumor-related deaths in developed nations [1]. About 78% of lung cancers belong to the category of non-small cell lung cancer (NSCLC). Further, NSCLC is pathologically divided into four groups: adenocarcinoma, squamous cell carcinoma (SCC), large cell carcinoma and neuroendocrine cancer [2]. Targeted molecular treatments have offered a number of appealing benefits to patients with adenocarcinoma [3–5]. However, these new therapies have brought not much effect in lung squamous cell carcinoma (LUSC) [6]. Thence, we need more valid treatments based on genomic methods for LUSC.

Plenty of studies have revealed that long noncoding RNAs (lncRNAs) may serve as crucial cis- or trans-regulators in multiple biological behaviors [2, 7]. Mutation and modulation of lncRNA expression will influence a large number of diseases. MicroRNA-205 host gene (MIR205HG) has been proofed to accelerate cell growth in Head and Neck Squamous Cell Carcinoma (HNSCC) by down-regulating miR-590-3p [8]. Nevertheless, studies on the function of MIR205HG in the progression of LUSC are still blank, so its biological functions are needed to be featured.

Recently, according to findings on a wide range of non-coding RNAs, microRNAs (miRNAs) are small non-coding RNAs participating in the suppression or down-regulation of target gene transcription in the way of sequence-dependent [1, 2]. The capacity of miRNAs is particular. A miRNA can control a great deal of protein-coding RNAs of human cells [9, 10]. Thence, regulating RNA networks may be interfered by the aberrant expression of miRNAs [11, 12], which is conducive to tumorigenesis, tumor metastasis and drug resistance [3, 4]. As a matter of fact, aberrantly expressed miRNAs have been studied in different cancers, including lung SCC [5, 13]. For instance, the expression levels of miR-133a were shown dramatically low in prostate cancer tissues compared with adjacent normal tissues [14]. MiR-299-3p has been explored to express aberrantly in certain types of cancers, such as colon carcinoma and lung cancer [15–17]. The exact role and functional mechanism of miR-299-3p in LUSC maintain uncovered. Thence, this study was designed to explore the molecular mechanism of relationship between MIR205HG and miR-299-3p in LUSC.

## Methods

### Cell culture

Human normal lung epithelial cell (BEAS-2B) and lung cancer cells (NCI-H520, SK-MES-1, NCI-H1703) were all bought from Chinese Academy of Sciences (Beijing, China). The above cells were inoculated in RPMI-1640 medium (Invitrogen, Carlsbad, CA, USA) adding 10% FBS (Invitrogen), then mixed with 1% penicillin/streptomycin (Sigma-Aldrich, Milan, Italy) and cultivated in a 5% CO_2_ incubator at 37 °*C. medium* for incubation was required to change every 3 days.

### Cell transfection

Cells were put into a fresh 6-well plate until cells were passaged at 80% confluence. NCI-H520 and SK-MES-1 cells were co-transfected with shRNAs targeting MIR205HG (sh-MIR205HG#1/#2) and sh-NC. The pcDNA3.1/MIR205HG, pcDNA3.1/MAP 3 K2 and the empty pcDNA3.1 were constructed by Genechem (Shanghai China). The miR-299-3p mimics and NC-mimics were also gained from Genechem. All transfections were conducted by Lipofectamine 2000 (Invitrogen).

### Quantitative real-time polymerase chain reaction (qRT-PCR) analysis

Total RNAs of cells were extracted by TRIzol reagent which was purchased from Invitrogen. The total RNAs were reverse-transcribed into cDNA under a Reverse Transcription Kit (Invitrogen). qRT-PCR analysis was achieved using SYBR Green Premix PCR Master Mix (Roche, Mannheim, Germany) under ABI HT9600 (Applied Biosystems, Foster City, CA, USA). The fold expression changes were calculated by using 2^-∆∆Ct^ method, and GAPDH/U6 was seen as the endogenous control.

### Colony formation assay

SK-MES-1 and NCI-H520 cells were plated into 6-well plates and cultured after transfection. After 14 days, cells were cleaned with PBS (Sigma-Aldrich) and fixed in paraformaldehyde (Sigma-Aldrich) for 10 min, dyed by crystal violet (Sigma-Aldrich) for 5 min. Finally, the visible colony numbers were calculated manually.

### Transwell migration assay

Serum-free medium containing SK-MES-1 and NCI-H520 cells was placed in the top compartment and the lower compartment was filled with 10% FBS (Sigma-Aldrich). Matrigel (Corning Incorporated, NY, USA) was additionally pre-coated on the apical compartment for migration assay. After cultivating for 48 h, the bottom of the chamber was fixed by methanol (Sigma-Aldrich), and then dyed with crystal violet (Sigma-Aldrich). The cells that had migrated were counted at 5 randomly chosen visual fields using an inverted microscope (Olympus, Tokyo, Japan).

### Western blot analysis

When reached to 80–90% confluent, cells were collected for total protein extraction by using RIPA cell lysis. After the concentration measured via a Pierce Bicinchoninic acid (BCA) Protein detection kit (Bio-Rad Laboratories, Hercules, CA, USA), proteins were isolated by performing SDS-PAGE and then moved onto a PVDF membrane (Beyotime, Shanghai, China). The membrane was sealed with 5% fat-free milk and rinsed by Tris-buffered saline/Tween 20 (TBST), followed by overnight incubation with the primary antibodies at 4 °C. The primary antibodies used in this study were as below: anti-E-cadherin (ab194982, Abcam), anti-N-cadherin (ab202030, Abcam), anti-Vimentin (ab193555, Abcam), anti-MAP 3 K2 (ab33918, Abcam) and antiGAPDH (ab8245, Abcam). Following washing in TBST, the membranes were subjected to 2 h of incubation with secondary antibodies at room temperature. The, the blots were visualized by the application of enhanced chemiluminescence (ECL; Amersham Pharmacia Biotec, Buckinghamshire, UK). GAPDH served as the internal control. The experiment was conducted in triplicate.

### Apoptosis analysis

Cell apoptosis was examined at 48 h after transfection using FITC Annexin V/PI Apoptosis Detection Kit I (Ribobio, Guangzhou, China). In short, cells were reaped and cleaned in PBS (Sigma-Aldrich) and resuspended in 70% cooled ethanol (Sigma-Aldrich). The apoptosis ratio was determined by using FlowJo software (Ashland, OR, USA).

### RNA immunoprecipitation (RIP) assay

RIP experiments were performed using the Magna RNA-Binding Protein Immunoprecipitation Kit (Millipore, MA, USA). Then, cell lysates were cultivated with RIP buffer containing magnetic beads conjugated with human anti-Ago2 (ab32381, Abcam) or anti-IgG (Millipore, MA, USA). Furthermore, qRT-PCR was performed to evaluate the expression levels of MIR205HG, miR-299-3p and MAP 3 K2. Normal IgG was considered as control.

### Luciferase reporter assay

The wild-type and mutant binding sites of MIR205HG or MAP 3 K2 to miR-209-3p were sub-cloned into pmirGLO dual-luciferase vector to construct plasmids and then co-transfected with miR-299-3p mimics and pcDNA3.1/MIR205HG into NCI-H520 or SK-MES-1 cells, separately. Dual-luciferase reporter system was applied for detecting the activity of luciferase.

### Statistical analysis

Data analysis was performed using GraphPad Prism 7 software package (La Jolla, CA, USA). Data came from at least three independent assays were showed as mean ± standard deviation (SD). ANOVA analysis and Student’s t-test were employed for evaluating difference of 2 or more groups. *P* < 0.05 had statistical significance.

## Results

### Cutting down MIR205HG expression impairs cell proliferation and migration abilities in LUSC

**Fig. 1:**
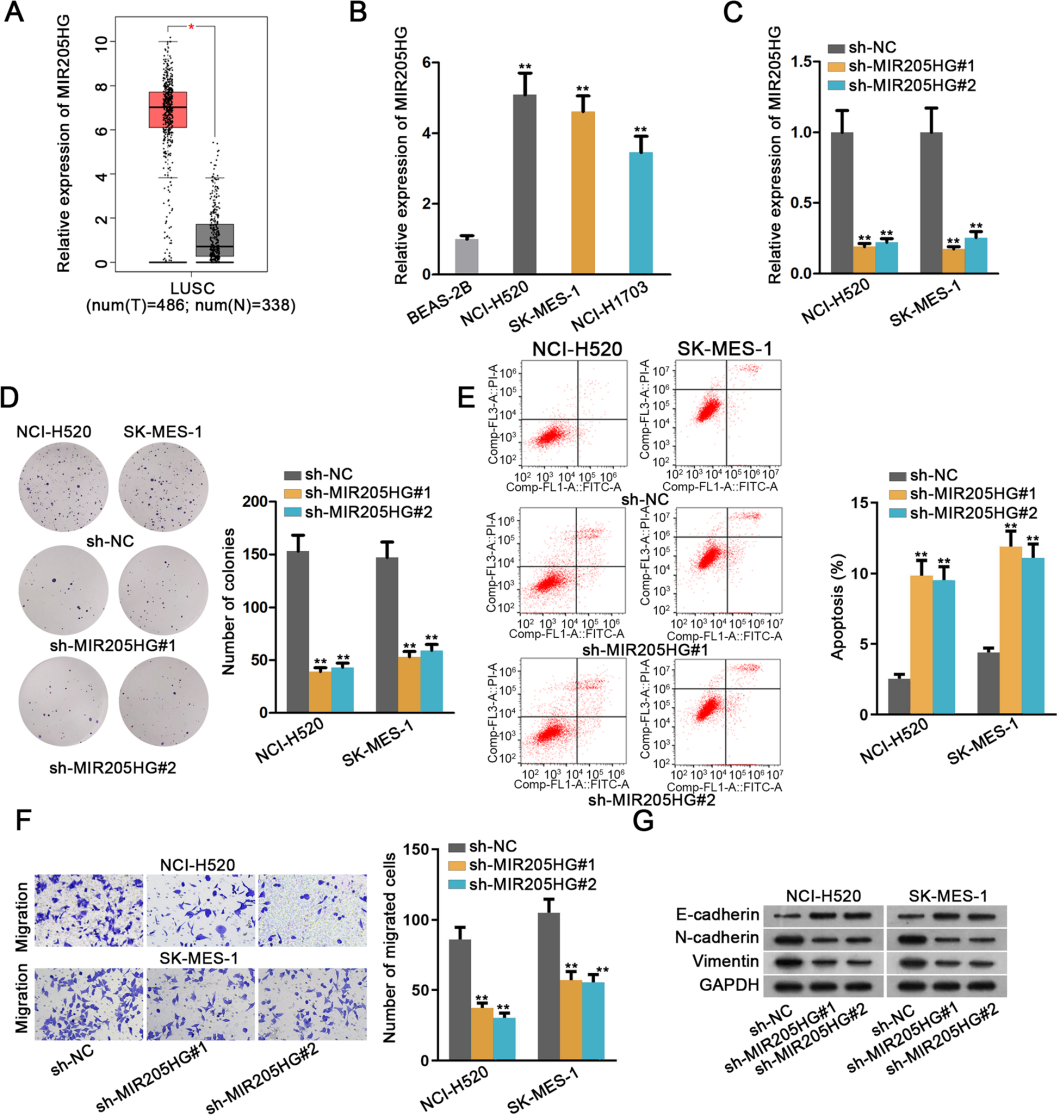
Cutting down MIR205HG expression impaired cell proliferation and migration abilities in LUSC. **a**. GEPIA dataset was applied to examine the relative expression of MIR205HG in LUSC tissues and adjacent normal tissues. **b**. Carrying out qRT-PCR to determine different expressions of MIR205H in normal lung squamous cell line (BEAS-2B) and LUSC cell lines (NCI-H520, SK-MES-1 and NCI-H1703). **c**. The relative expression of MIR205H under the condition of down-regulating MIR205H was measured by qRT-PCR analysis. **d**. Number of colonies was assessed by colony formation assay to detect cell proliferation ability. **e**. Cell apoptosis rate was estimated by flow cytometry analysis. **f**. Transwell assay was used to measure cell migration capability by counting number of migrated cells. **g**. The expressions of the epithelial marker (E-cadherin) and the mesenchymal markers (N-cadherin and Vimentin) were measured by western blot analysis. All data were displayed as the mean ± SD. *P < 0.05, **P < 0.01

MicroRNA-205 host gene (MIR205HG) has been proofed to accelerate cell growth in HNSCC by down-regulating miR-590-3p [8]. Here in the first place, we applied GEPIA dataset to examine expression of MIR205HG in LUSC tissues. The results depicted that MIR205HG was significantly up-regulated in LUSC tissues compared with the adjacent normal tissues (Fig. 1a). Then, qRT-PCR detected that MIR205HG expression was relatively high in LUSC cell lines (NCI-H520, SK-MES-1 and NCI-H1703) compared with the normal control (Fig. 1b). Besides, NCI-H520 and SK-MES-1 cells were singled out for the forthcoming experiment for that they the higher level of MIR205HG.

Then, two shRNAs targeting MIR205HG were transfected into NCI-H520 and SK-MES-1 cells to detect the efficiency of down-regulating MIR205HG. Consequently, as depicted in Fig. 1c, the expression level of MIR205HG in selected cell lines were decreased conspicuously. Functionally, number of colonies declined distinctly in response to MIR205HG down-regulation (Fig. 1d). On the contrary, cell apoptosis ability was enhanced obviously, as demonstrated in Fig. 1e. In parallel, transwell assay showed that migration abilities of both cell lines were impaired observably (Fig. 1f). Moreover, from the results of western blot analysis, the expression of the epithelial marker (E-cadherin) was elevated observably while that of the mesenchymal markers (N-cadherin and Vimentin) was reduced comparably (Fig. 1g, Supplementary Figure 1A and Supplementary information file 1A). The above findings illustrated that MIR205HG had tumor-promoting function in LUSC.

### MIR205HG is an upstream factor for miR-299-3p in LUSC

**Fig. 2:**
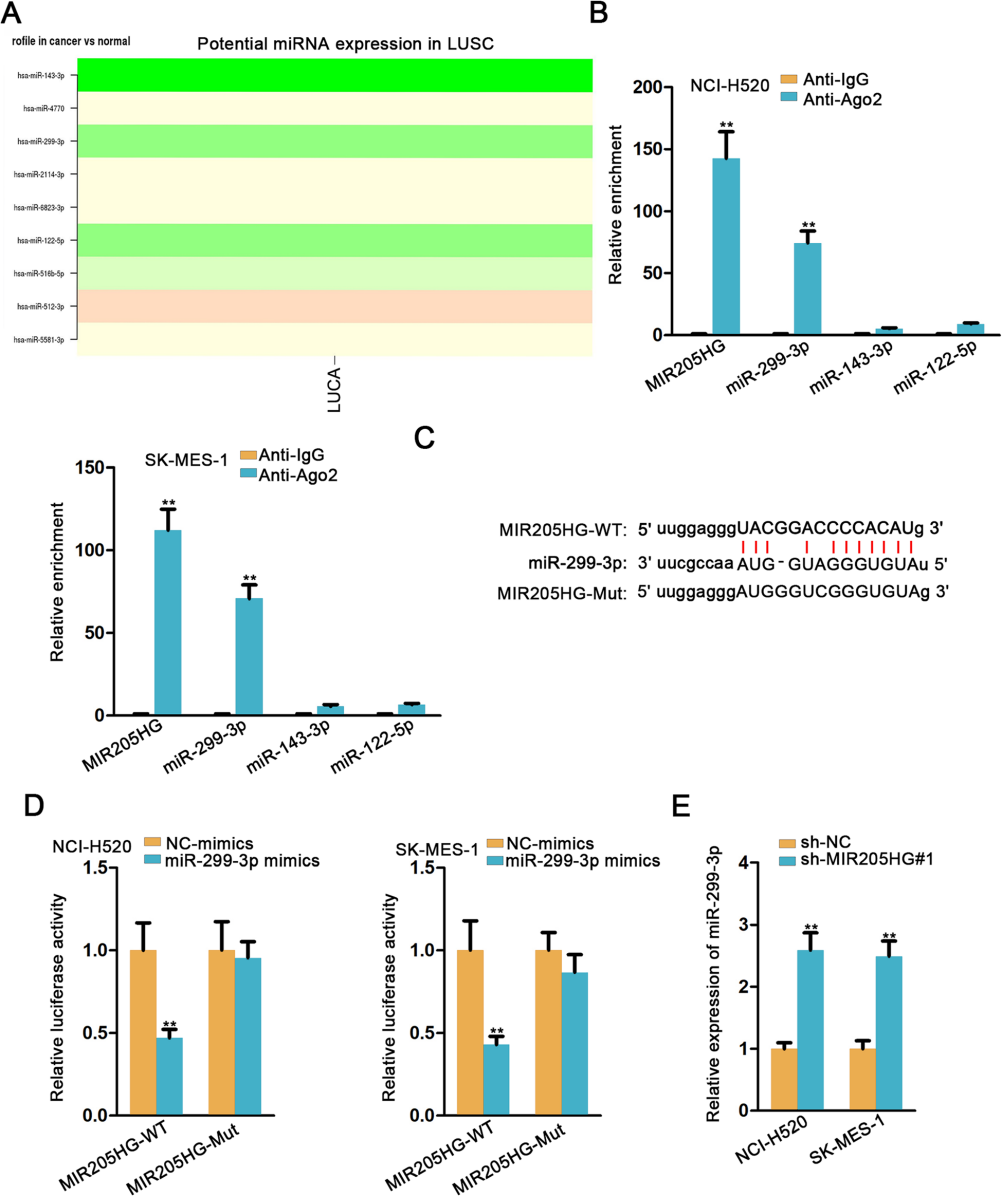
MIR205HG was an upstream factor for miR-299-3p in LUSC. **a**. The potential miRNA expression in LUSC was downloaded from dbDEMC 2 website. **b**. RIP assay was applied to confirm the relationship between MIR205HG and miR-299-3p. **c**. StarBase database was used to predict the potential binding sites between MIR205HG and miR-299-3p. **d**. Luciferase reporter assay was carried out to verify that MIR205HG could bind with miR-299-3p. **e**. qRT-PCR analysis was performed to assess the relative expression of miR-299-3p in both cell lines transfected with sh-MIR205HG#1. All data were showed as the mean ± SD. **P < 0.01

We obtained potential miRNAs which had binding sites with MIR205HG via starBase database. Then through searching out the expression level of candidate miRNAs in LUSC from dbDEMC 2 website, 3 miRNAs (miR-299-3p, miR-143-3p and miR-122-5p) with low expression were selected out (Fig. 2a). RIP assay was applied to verify the binding situations between 3 miRNAs and MIR205HG. As denoted in Fig. 2b, only miR-299-3p and MIR205HG were significantly enriched in Ago2 precipitated RNA-induced silencing complexes (RISCs), indicating that miR-299-3p and MIR205HG involved in ceRNA axis. It further bolted miR-299-3p as our target miRNA. Figure 2c depicted the binding sites between miR-299-3p and wild type MIR205HG (MIR205HG-WT)/mutant MIR205HG (MIR205HG-Mut). Luciferase reporter assay exhibited that the luciferase activity of MIR205HG-WT obviously declined by elevated miR-299-3p overexpression, whereas that of MIR205HG-Mut bore little alteration (Fig. 2d). Besides, the expression of miR-299-3p was up-regulated dramatically under the condition of depleting MIR205HG in both cell lines (Fig. 2e). Hence, we concluded that miR-299-3p was the downstream molecule of MIR205HG in LUSC.

### MAP 3 K2 is the target gene of miR-299-3p and indirectly regulated by MIR205HG

**Fig. 3:**
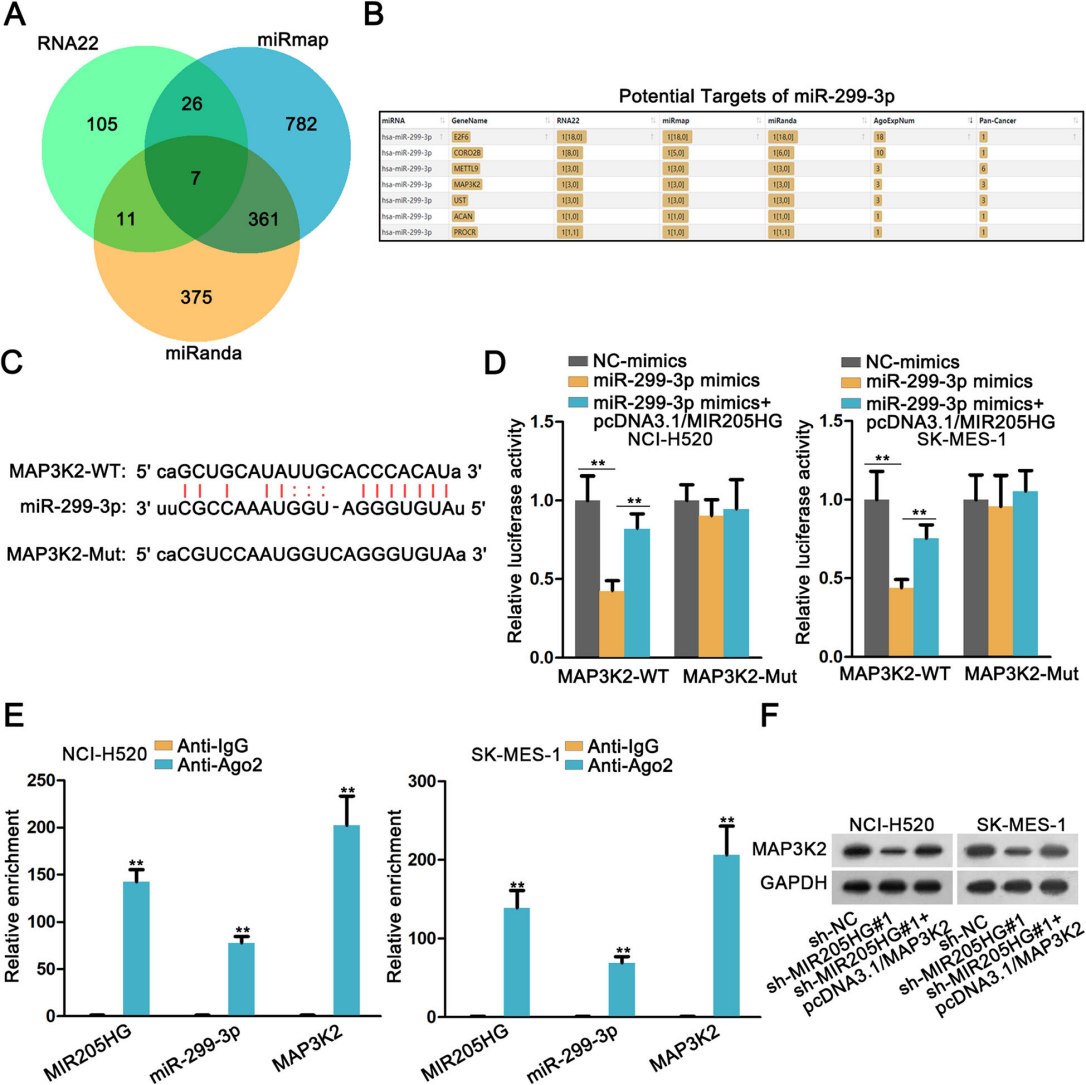
MAP 3 K2 was the target gene of miR-299-3p and indirectly regulated by MIR205HG. **a**. Venn data was used to exhibit the common target mRNAs from 3 databases (RNA22, miRmap and miRanda). **b**. The potential targets of miR-299-3p which was downloaded from starBase website. **c**. StarBase database showed the binding sites between MAP 3 K2 and miR-299-3p. **d**. The binding interaction between MAP 3 K2 and miR-299-3p was further assessed by luciferase reporter assay. **e**. RIP assay was performed to confirm that MIR205HG, miR-299-3p and MAP 3 K2 co-existed in RISCs. **f**. Western blot analysis was used to determine the protein expression level of MAP 3 K2 in cell lines transfected with sh-MIR205HG#1 and pcDNA3.1/MAP 3 K2. All data were expressed as the mean ± SD. **P < 0.01

To forecast the possible target of miR-299-3p, we acquired the common target genes from 3 databases, including RNA22, miRmap and miRanda (Fig. 3a). There were seven candidate target genes of miR-299-3p (Fig. 3b). Previous studies have reported that MAP 3 K2 acted as an oncogene in the progression of certain types of cancer [18, 19]. Thus, MAP 3 K2 was chosen as the target gene of miR-299-3p. Simultaneously, miR-299-3p and MAP 3 K2 had binding sites, which were displayed in Fig. 3c. Luciferase reporter assay was used to further prove binding relationship between miR-299-3p and MAP 3 K2. As illustrated in Fig. 3d, miR-299-3p overexpression decreased the luciferase activity of MAP 3 K2-WT group via binding to MAP 3 K2-WT, while the luciferase activity of mutant MAP 3 K2 (MAP 3 K2-Mut) group had no alteration. Similarly, RIP assay showed that MIR205HG, miR-299-3p and MAP 3 K2 were all enriched in Ago2 containing beads in both cell lines (Fig. 3e). Via western blot analysis, the expression level of MAP 3 K2 was decreased with the down-regulation of MIR205HG, then increased with the overexpression of MAP 3 K2 (Fig. 3f, Supplementary Figure 1B and Supplementary information file 1B). To draw a conclusion, MAP 3 K2 acted as the target gene of miR-299-3p and indirectly regulated by MIR205HG.

### MIR205HG accelerates LUSC development by regulating miR-299-3p/MAP 3 K2 pathway

**Fig. 4:**
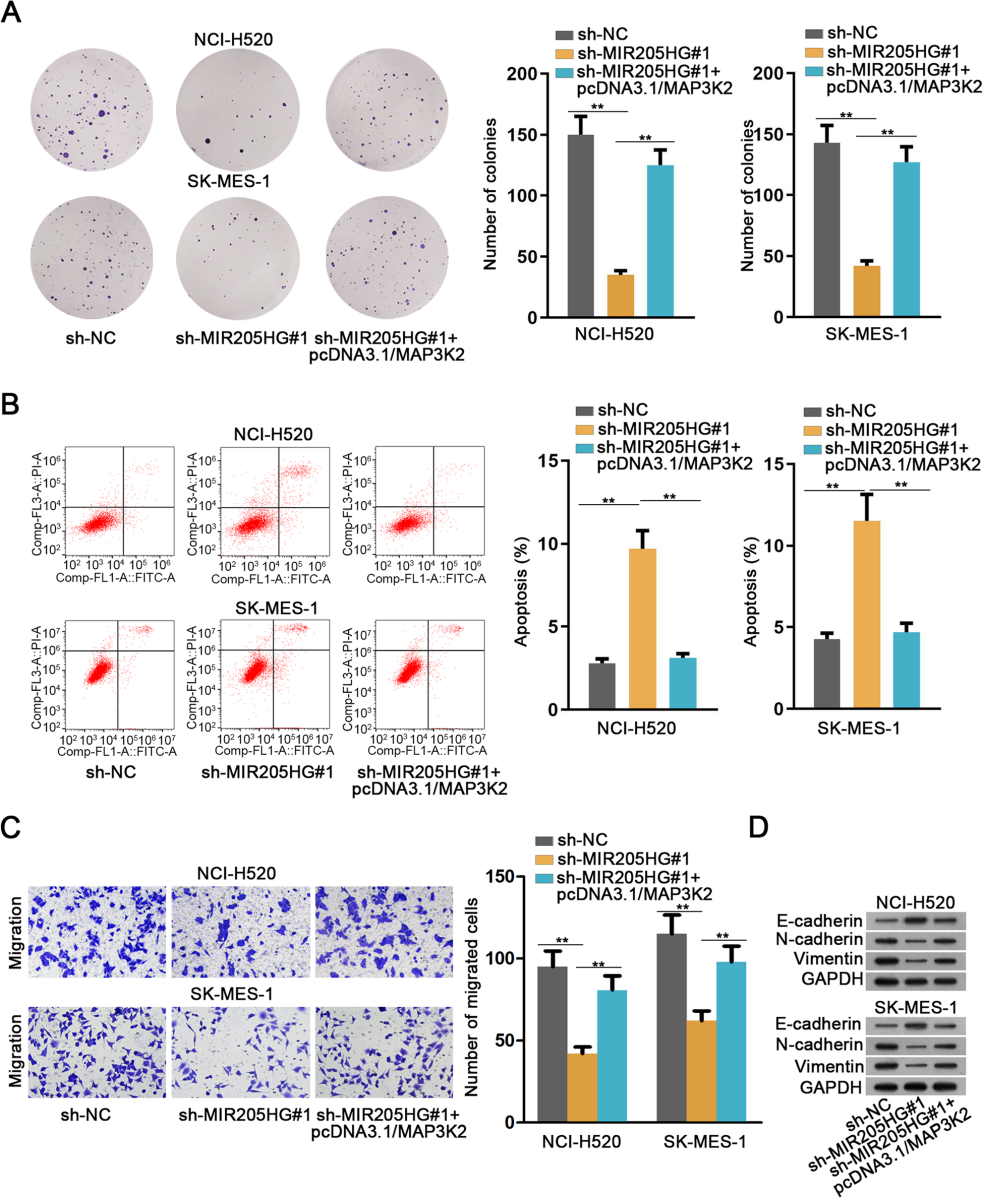
MIR205HG accelerated LUSC development by modulating miR-299-3p/MAP 3 K2 signaling. **a**. Colony formation assay was applied to detect cell proliferation capacity in both cells by transfection with sh-MIR205HG#1 and pcDNA3.1/MAP 3 K2. **b**. Cell apoptosis rate was tested via flow cytometry analysis. **c**. Cell migration capacity was estimated through conducting transwell assay. **d**. EMT-related protein level was evaluated by western blot analysis. All data were expressed as the mean ± SD. **P < 0.01

We carried out rescue function assays to verify the effects of modulating relationship between MIR205HG and MAP 3 K2 on LUSC development. The results revealed that overexpressing MAP 3 K2 could restore number of colonies reduced by reducing MIR205HG (Fig. 4a). In contrast, the overexpression of MAP 3 K2 decreased cell apoptosis up-regulated by MIR205HG knockdown (Fig. 4b). Transwell assay also proved that elevating MAP 3 K2 could recover the reduced cell migration abilities caused by silenced MIR205HG (Fig. 4c). Evidence from western blot analysis suggested that overexpression of MAP 3 K2 decreased the expression of E-cadherin that was up-regulated by MIR205HG knockdown. N-cadherin and Vimentin expressions which were decreased by MIR205HG knockdown were enhanced by overexpression of MAP 3 K (Fig. 4d, Supplementary Figure 1C and Supplementary information file 1C). Altogether, MIR205HG could up-regulate MAP 3 K2 expression by sponging miR-299-3p in LUSC, thereby regulating LUSC cell proliferation, apoptosis, migration and EMT process.

## Discussion

This study indicated that MIR205HG may be a crucial regulator for cell proliferation, migration and EMT progression in LUSC. First of all, we found that the expressions of MIR205HG in LUSC tissues and cell lines (NCI-H520, SK-MES-1 and NCI-H1703) were highly expressed. In addition, down-regulation of MIR205HG suppressed cell proliferation, migration and EMT process, whereas enhanced cell apoptosis. Besides, the results also revealed that reducing MIR205HG increased the expression level of miR-299-3p. It further proved that MIR205HG was an upstream factor for miR-299-3p. On the other hand, MAP 3 K2 acted as the target gene of miR-299-3p, which was regulated by MIR205HG indirectly. Increasing MAP 3 K2 expression could restore the effects caused by inhibiting MIR205HG on cell biological functions. To sum up, MIR205HG could regulate MAP 3 K2 expression via miR-299-3p to promote the progression of LUSC. Furthermore, evidence of MIR205HG functionality in LUSC might extend to clinical pathologic features of LUSC patients. This study may have some referential meaning for the development of possible therapeutics against LUSC.

It is well-acknowledged that lncRNAs have multiple molecular functions, including adjusting transcriptional forms and protein activities and altering RNA processes [20, 21]. However, it is still not clear whether these lncRNAs are the causes or symptoms of morbid state. A vast number of studies have indicated that many disordered lncRNAs existed in all types of human diseases, including cancers [22, 23]. This research demonstrated the high expression of MIR205HG in LUSC tissues and cell lines. Depletion of MIR205HG repressed cellular activities in LUSC, suggesting that MIR205HG worked as an oncogene in LUSC.

Generally, miRNAs play a role in cancer cells by means of promoting degradation of the targeted genes [24, 25]. ABCE1 is the target gene of miR-299-3p in lung cancer cells [16]. In human colon carcinoma, miR-299-3p suppresses the tumor process in the way of immediately targeting VEGFA [15]. Then we intended to probe a new target of miR-299-3p in LUSC cells. In this study, MAP 3 K2 was identified as the target mRNA of miR-299-3p. Overexpressing MIR205HG could cut down miR-299-3p expression through binding with it. In the same time, MIR205HG enhanced MAP 3 K2 expression via sequestering miR-299-3p.

Previous papers found out that MAP 3 K2 could distinguish tumor cells from non-tumor normal cells [18]. Furthermore, MAP 3 K2 contributes to the growth and migration of liver cancer cells [24, 25]. MiR-520b targeting MAP 3 K2 could impair hepatoma cell proliferation ability [19]. These findings all revealed that MAP 3 K2 probably play a significant role in the development of liver cancer. Similarly, MAP 3 K2 in this research was proved to exert tumor-promoting effects in LUSC. In rescue function assays, up-regulating MAP 3 K2 expression recovered cell proliferation and migration abilities and EMT process, which were impaired by depleting MIR205HG.

## Conclusions

MIR205HG up-regulates MAP 3 K2 expression via sponging miR-299-3p to facilitate cell proliferation, migration and EMT process in LUSC.

## Supplementary information

**Additional file 1: Figure S1.** A. Another two data obtained from two repeated western blot analyses for Fig. 1g. B. Another two data obtained from two repeated western blot analyses for Fig. 3f. C. Another two data obtained from two repeated western blot analyses for Fig. 4d.

**Additional file 2.** A. The original western blot data for corresponding cropped data in Fig. 1g and Supplementary Fig. 1A. B. The original western blot data for corresponding cropped data in Fig. 3f and Supplementary Fig. 1B. C. The original western blot data for corresponding cropped data in Fig. 4d and Supplementary Fig. 1C.

## Abbreviations

LUSC: Lung squamous cell carcinogenesis
lncRNA: Long non-coding RNA
EMT: Epithelial-to-mesenchymal transition
ceRNA: Competing endogenous RNA
NSCLC: Non-small cell lung cancer
SCC: Squamous cell carcinoma
MIR205HG: microRNA-205 host gene
HNSCC: Head and neck squamous cell carcinoma
miRNA: microRNA
mRNA: Messenger RNA
WT: Wild type
Mut: Mutant
RIP: RNA immunoprecipitation
MAP 3 K2: Mitogen-activated protein kinase kinase kinase 2
qRT-PCR: Quantitative real-time polymerase chain reaction
NC: Negative control
cDNA: Complementary DNA
PBS: Phosphate buffer saline
FBS: Fetal bovine serum
SDS-PAGE: Sodium dodecylsulphate polyacrylamide gel electrophoresis
PVDF: Polyvinylidene fluoride
SD: Standard deviation
ANOVA: Analysis of variance
RISC: RNA induced silence complex
ABCE1: ATP binding cassette subfamily E member 1
VEGFA: Vascular endothelial growth factor A
