# Supplementary figures and images for: MIR205HG acts as a ceRNA to expedite cell proliferation and progression in lung squamous cell carcinoma via targeting miR-299-3p/MAP3K2 axis
Source: BMC Pulm Med. 2020 Jun 8;20:163. doi: 10.1186/s12890-020-1174-2 (PMC7278044; doi:10.1186/s12890-020-1174-2)

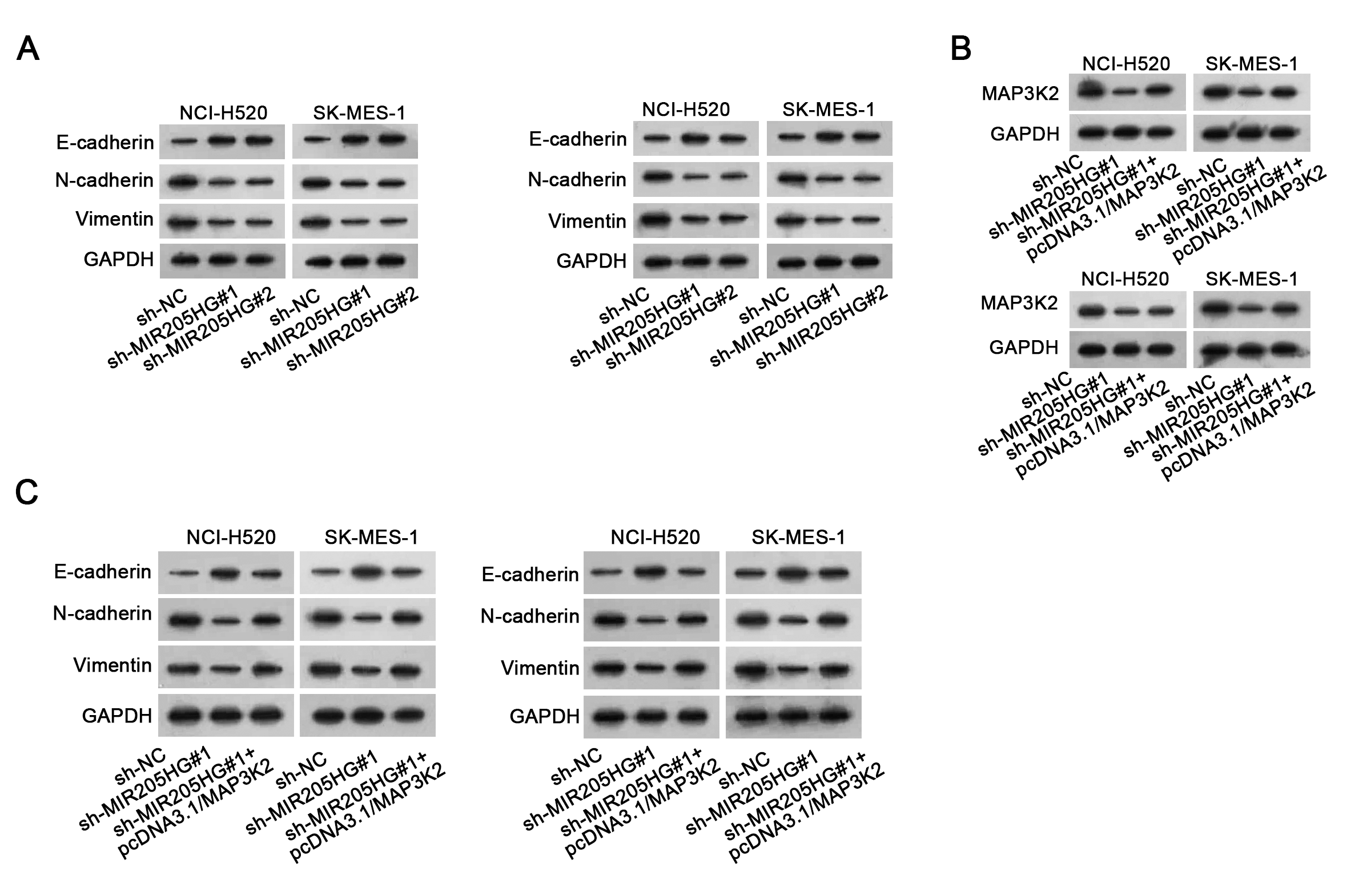

Supplement: Supplementary file 1 — Additional file 1: Figure S1. A. Another two data obtained from two repeated western blot analyses for Fig. 1g. B. Another two data obtained from two repeated western blot analyses for Fig. 3f. C. Another two data obtained from two repeated western blot analyses for Fig. 4d. [file 12890_2020_1174_MOESM1_ESM.tif]

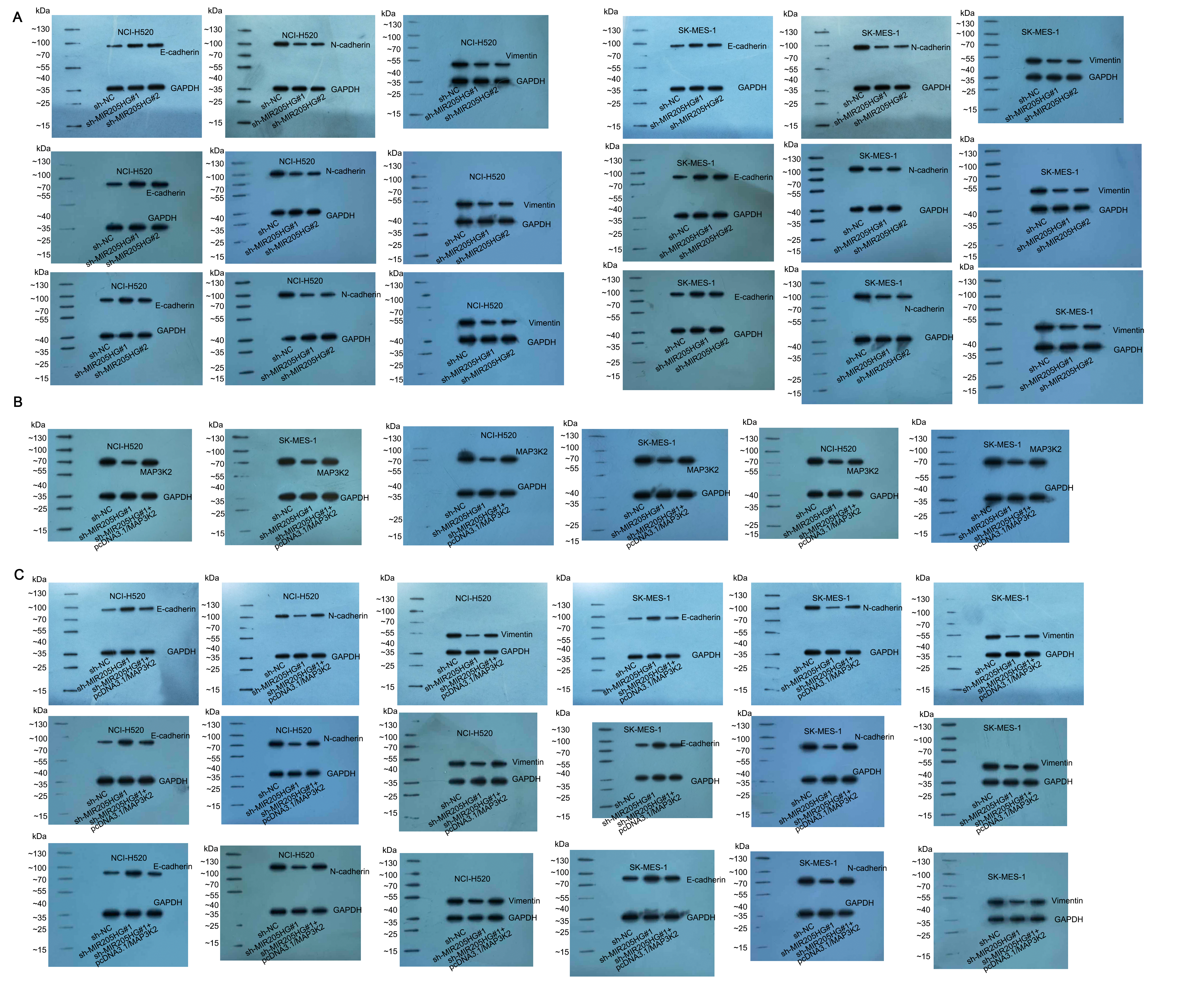

Supplement: Supplementary file 2 — Additional file 2. A. The original western blot data for corresponding cropped data in Fig. 1g and Supplementary Fig. 1A. B. The original western blot data for corresponding cropped data in Fig. 3f and Supplementary Fig. 1B. C. The original western blot data for corresponding cropped data in Fig. 4d and Supplementary Fig. 1C. [file 12890_2020_1174_MOESM2_ESM.tif]
